# Barriers to the Right to Health Among Patients of a Public Emergency Department After Implementation of the Affordable Care Act

**DOI:** 10.1089/heq.2018.0071

**Published:** 2019-05-02

**Authors:** Shamsher Samra, Elizabeth Pelayo, Mark Richman, Maureen McCollough, Breena R. Taira

**Affiliations:** Department of Emergency Medicine, Olive View-UCLA Medical Center, Sylmar, California.

**Keywords:** ACA, Affordable Care Act, health care disparities, immigrant, insurance, right to health

## Abstract

**Purpose:** Emergency physicians are witnesses to the impact of socioeconomic determinants of health on physical and psychiatric illness. Understanding structural barriers to the right to health (RTH) serves as a foundation for interventions to promote health equity. This study was performed to determine self-described barriers to fulfillment of the RTH among a public emergency department (ED) patient population.

**Methods:** A convenience sample survey between June and August 2014 of 200 patients in public ED assessing demographic characteristics and desired assistance with 36 barriers to fulfillment of the RTH.

**Results:** There was a high demand for specialty care (91%, 182/200), access to primary care (87.5%, 175/200), and access to health insurance (86%, 172/200). Undocumented residents were significantly more likely to cite health insurance as the most important area for assistance (*p*=0.04).

**Conclusion:** Despite implementation of Affordable Care Act, access to health care and insurance were still perceived as the most important barriers among underserved patient populations, particularly undocumented groups.

## Introduction

Signed into law March 30, 2013, The Patient Protection and Affordable Care Act (ACA) sought to improve access to, financial coverage of, and quality of health care by decreasing the number of uninsured Americans through the expansion of public and private health insurance, while decreased health care costs. The ACA was anticipated to reduce the number of uninsured Americans by 30 million by 2016, marking one of the largest expansions of health insurance in the United States history, through a combination of an individual mandate to have health insurance, expansion of Medicaid to legal residents <138% of the federal poverty level (FPL), and subsidies to participate in health insurance exchanges for those between 100% and 400% of the FPL.^1^

By June 2015, the estimated number of uninsured Americans had decreased by 16.9 million.^2^ Of note, large segments of the populations fall outside the purview the ACA, including those that are explicitly excluded, such as the undocumented or incarcerated, and those that qualify for exemptions to the mandate.^3^

Despite this expansion of health care insurance, barriers to health insurance, health care, and health, even among those not explicitly excluded from the law, remain.^4^ These persistent barriers to health, despite normative advances in health care access, not only suggest incomplete implementation of the ACA, but also persistent social and structural barriers to health. In 1976, Thomas McKeown put forth the thesis that health care, and even public health, contributed little to the advances in life expectancy in the 19th and 20th centuries, it instead attributing gains to improvements in quality of life and nutrition. Although the interpretation remains controversial, the idea that there are powerful social determinants of health such as social class, employment status, education, and stress has become mainstream.^5^

For emergency physicians, the relationship between patients' health outcomes and forces that extend beyond the walls of hospitals, outpatient clinics, and the emergency department (ED) is obvious. Emergency physicians are arguably among those most proximate to the acute health manifestations of the chronic structural barriers of poverty, illiteracy, homelessness, and political disenfranchisement. This proximity, combined with the unique role of EDs as “the only component of the entire social welfare system that is protected by law for many of the most disadvantaged” makes it a unique space both to understand and address barriers to health.^6^

The purpose of this study was to evaluate barriers to health among patients presenting to an urban ED. We intentionally use health and human rights language as a normative framework that defines health as “complete physical, mental and social well-being and not merely the absence of disease or infirmity.”^7^ This definition of health is wedded to the human rights perspective that every individual is entitled to the “the highest attainable standard of physical and mental health,”^8^ which necessitates access to health care, not simply health insurance. The framework further recognizes the importance of equity and the impact of social and economic conditions on individual health.^9^

Alternative frameworks, such as “patients' rights” or “consumer rights,” fail to encompass the full range of rights that derive from inherent human dignity.^10^ “Patients' rights” are limited to the interaction between a patient and the health system and include rights to privacy/confidentiality, quality, autonomy, and informed consent. “Consumer rights” derive from the transactional relationship between the patient and the health system and are similar to contractual rights. Because determinants of health include “social determinants” beyond what occurs in the context of the patient–health system interaction, patients' or consumer rights do not address the full scope of the right to health (RTH). This study aims to identify self-reported barriers (including housing, food security, and personal safety) to health among a safety-net ED population shortly after implementation of the ACA. Consequently, the more expansive “right to health” lens was utilized.

## Methods

### Design

This study utilized a voluntary, anonymous questionnaire. All interviews were conducted within the ED between June and August 2014, ∼2 months after the March 31, 2014 registration deadline for the ACA initial enrollment period. All interviews were conducted during daytime hours between 8 AM and 5 PM.

### Setting

All interviews were conducted within the ED of the primary safety-net hospital in Northern Los Angeles County. The hospital was a 377-licensed bed, publicly supported, academic teaching hospital. It was staffed with 200 beds and served a medically indigent population. The hospital had an annual ED census of ∼54,000 visits and admitted ∼15,000 patients per year. Fewer than 5% of patients have private insurance; the remainder were uninsured or have Medicaid (“Medi-Cal” in California). All interviews were conducted within patient rooms after triage and a rapid medical screening examination.

### Study population

A convenience sample of 245 patients were selected by approaching those either waiting to be seen or pending reevaluation by their care provider. The study excluded those younger than 18 years of age or who did not speak either English or Spanish, and those who were unresponsive or deemed to be in distress. Informed consent was obtained from all respondents.

### Assessment

#### Measures and outcomes

To assess barriers to the RTH, participants were asked face-to-face through an anonymous, structured questionnaire whether they would desire assistance relating to seven domains of socioeconomic determinants of health: health care, housing security, food security, physical security, financial security, family care, and the community environment. These seven domains were chosen based on published social determinants of health.^11,12^

The domains were further subdivided into 36 issues: health insurance, primary care, specialty care, counseling, medical debt, access to rehabilitation or detox facilities, transportation to and from medical facilities, medication storage, housing conditions, obtaining or keeping housing, issues with a landlord, paying for utility bills, foreclosure, food security, locations to purchase healthy foods, personal safety, family violence, neighborhood violence, financial concerns, employment concerns, disability benefits, public benefits, English classes, adult literacy programs, financial planning, debt relief, youth programs, educational issues, family legal concerns, elder care, child care, pet care, criminal justice, immigration, and community improvement. Participants were asked to select the single most pressing area of concern at the time from among the 36 issues.

The questionnaire also assessed demographic characteristics, including age, gender, zip code, household size, citizenship status, and type of health insurance. There was a question regarding the need for legal services within the last year. Finally, the questionnaire assessed self-reported proxies of socioeconomic status including literacy, income, level of education, employment status, and health insurance status.

The questionnaire was pilot tested on patients and consequent revisions made before adopting the final version. For data collection, the questionnaire was administered by one female, native Spanish-speaking research assistant volunteer trained by the ED Research Director in research interviewing technique and who had no previous relationship to study subjects before study commencement. The research assistant explained the intent of the study and her interest in exploring social determinants of health among this population. She read questionnaire items to the patients and marked their responses on the form. No other persons were present with subjects during questionnaire completion.

Questionnaires were generally completed within 15 min. All questionnaires were completed in full. No audio or visual recordings were made, and no field notes were kept. Data saturation techniques were not utilized. No repeat interviews were performed. Participants did not provide feedback on the findings.

### Statistical analysis

Questionnaires were collected on paper and managed using REDCap electronic data capture tools hosted at Olive View-UCLA Medical Center. Data entry was performed by the research assistant. Data were then exported into Stata version 10 for analysis (College Station, TX). The data were analyzed using Stata and descriptive statistics were generated including frequencies and percentages for categorical variables. Univariate analyses were also performed to evaluate associations between certain demographic characteristics and any of the 36 barriers to the RTH.

Institutional Review Board approval was obtained from the Olive View-UCLA Education and Research Institute.

## Results

### Demographics

Of 245 potential participants approached, 200 participated in the survey. The majority of respondents were women (61%, 122/199); mean age was 45 years (standard deviation [SD]=15 years). Levels of English ability and literacy were low; 65% (128/196) endorsed Spanish as their primary language; 55% (108/195) self-identified as being literate in English; and 7% (13/195) responded as being nonliterate in their primary language. Similarly, levels of formal education were low. Only slightly more than half (53%, 105/199) of respondents had completed a high school level of education.

The questionnaire identified high levels of poverty, with 72.6% (135/186) of respondents reporting a household income <$20,000/year, with a mean household size of 3.2 individuals (SD=1.9). Only 12.5% (25/198) of respondents were engaged in full-time, year-round work. More than one-third respondents (36.5%, 70/196) were undocumented. When asked to describe their health insurance status, 40% (80/199) responded “no insurance,” 36% (71/99) endorsed either Midicare or full-scope Medi-Cal, 14% (28/199) stated “emergency Medi-Cal,” and 9% (18/99) endorsed “county coverage” (e.g., sliding scale; Table 1).

**Table 1.** Demographic Characteristics

Age (years), mean (SD)	45 (15)
Gender (%)	
Male	39
Female	61
Occupants per household (mean)	3.2
Citizenship status (%)	
U.S. citizen	39
Undocumented	36
Legal permanent resident	16
Other (visa, DACA, temporary protected status, asylee/refugee)	9
Health insurance status (%)	
No insurance	40
Medicaid (“Medi-Cal”)/Medicare	36
Emergency Medi-Cal	14
Other (county coverage, private, Covered California Insurance Exchange)	11
Education level (%)	
Less than high school	47
Completed high school	20
Some college	16
Completed college	17
Employment status (%)	
Unemployed	21
Full-time, year round	13
Disabled	12
Retired	5
Other (seasonal, homemaker, part-time)	49
Primary language (%)	
Spanish	65
English	28
Other	7
English literacy (%)	55
Literacy in native language	93
Annual household income (%)	
<$10,000	42
$10,000–$19,999	30.7
20,000–$49,999	25.3
$50,000–$99,999	1.6
≥$100,000	0.5

DACA, Deferred Action for Childhood Arrivals; SD, standard deviation.

### Barriers to the RTH

Among study participants, the vast majority indicated desire for assistance with health care-related services, including assistance seeking specialty care (91%, 181/199), primary care (88%, 175/200), and health insurance (86%, 172/200). Although health care was the most frequently cited area of concern, there was also a high prevalence of concern with many determinants of health, including food, employment, and housing insecurity; the community environment; transportation; and medical debt (Table 2).

**Table 2.** Endorsed Need in Addressing Barriers to Health

Barriers to health	Yes (%)
Specialty care	91.0
Primary care	87.5
Health insurance	86.0
Medical debt	76.9
Place to buy healthy food	70.5
Transport to health care facilities	69.0
Counseling	68.0
Community improvement	66.8
Keeping housing	63.8
Financial concerns	60.5
Employment concerns	60.5
Public benefits	60.5
Paying for utility bills	59.3
English classes	57.8
Disability benefits	55.8
Youth programs	55.3
Neighborhood violence	54.3
Immigration	54.3
Food security	53.8
Financial planning	51.5
Housing conditions	51.3
Obtaining housing	50.0
Rehab/Detox	48.0
Debt relief	47.7
Educational issues	47.7
Medication storage	47.0
Elder care	43.0
Child care	43.0
Issues with landlord	41.7
Family legal concerns	39.7
Family violence	39.2
Adult literacy programs	38.7
Criminal justice	38.5
Personal safety	37.5
Foreclosure	33.7
Pets	31.5

When asked what was the single most pressing issue they faced at the time of the interview, participants most frequently cited health insurance (30%, 59/196) followed by concerns regarding immigration (12%, 23/196) and primary care (9%, 18/196; Table 3). Chi-square analysis between demographic characteristics and the prevalence of endorsed need suggest those who self-identified as being undocumented were significantly more likely to endorse health insurance as their most important need (*p*=0.04).

**Table 3.** Most Important Expressed Area of Need

Area of need	%
Access to health insurance	30.1
Immigration assistance	11.7
Access to primary care	9.2
Housing	4.6
Access to specialty care	0.7
Disability benefits	0.6
Medical debt	0.4
Other	31.8

## Discussion

The results of this study demonstrate that, shortly after the implementation of the ACA, barriers to both health and health care persisted among patients of a safety-net health institution. There was high level of demand for assistance in obtaining health insurance, primary care, and specialty care, with almost 50% of the participants endorsing these issues as their most important need at the time of the interview. Concerns regarding access to health care were especially marked among those who self-identified as undocumented. Furthermore, the study demonstrates vulnerability among this patient population with high rates of poverty, unemployment, housing insecurity, and illiteracy.^13^ As an example, 72.6% of the study population reported an annual household income <$20,000 dollars, in comparison with median household income of $56,000 for Los Angeles County and $53,000 nationally from 2009 to 2013.^14^ Similarly, 36.5% of participants self-reported as being undocumented, and undocumented individuals are estimated to make up 10% of the population in Los Angeles County and ∼4% of the national population.^15^

Although this study was not designed to fully delineate the persistent barriers to health care shortly after the ACA implementation, the data point toward a multifactorial explanation. Our results can be partially explained by the exclusion of undocumented individuals from the ACA. Although the 35% rate of undocumented observed in this study is not representative of the broader national picture, it is estimated that there are >12 million undocumented individuals residing within the United States.^12^ Exclusion from health insurance contributes to increased dependence on the ED for medical care, a lack of preventative services, delayed management of illness, and the “cycle of preventable hospitalizations.”^16^ Although this decision has been subject to controversy and debate, it is inconsistent with a value system that recognizes the RTH.

The conflict of dual loyalty faced by practitioners who aim to serve patients equally but work as a part of a health system with structural (including legal) barriers to health care and health has been previously described and is cited as a common element for the violation of the RTH in both “open” and “repressive” societies.^10^ Moving forward, efforts to expand health insurance coverage to the undocumented should be considered to overcome this barrier. Similarly, ∼4 million people are excluded because of their state's decision not to expand Medicaid as a part of the ACA, including ∼3 million in Texas and Florida alone.

Furthermore, our data demonstrate significant barriers to health and health care access even for those populations not explicitly excluded from the ACA. Likely contributing to this patient population's inability to actualize their access to health care are social and economic factors such as low levels of education, lack of transportation, limited English proficiency, poverty, and employment insecurity. These barriers disproportionately affect the underserved and pose significant barriers to health care. Sociologists use the term “structural vulnerability” to describe “a positionality that imposes physical/emotional suffering on specific population groups and individuals in patterned ways” that is borne out of “class-based economic exploitation and cultural, gender/sexual, and racialized discrimination.”^10^ Just as natural disasters are far from random, disproportionately affecting those with housing and economic insecurity, medical emergencies often disproportionately affect populations affected by structural vulnerability. Health care systems seeking to fulfill the RTH among the poor must take these factors into account. Given the ED's unique role in the social welfare system, it may serve as the ideal venue to both screen for and address social and economic barriers to health care, in cooperation with community-based organizations.

Third, this study suggests a need to ensure funding and quantity of services be afforded to populations historically served by the safety-net health system to overcome residual barriers to health care. A large percentage of the insured study cohort endorsed concerns regarding access to primary and specialty care, suggesting a lack of resources to serve the expanded number of insured patients. This hypothesis is supported by national data demonstrating a lack of primary care doctors to serve the expanding population of insured individuals and the cutbacks in intended funding to community clinics as a part of the ACA.^17^ As a result, the theoretical opportunity for health care diverges from the actual access to health care for large segments of the populations served at safety-net health facilities. Given this discrepancy, caution must be taken in withdrawing funding from the traditional safety-net system and hospitals serving a disproportionate share of uninsured and underinsured patients (i.e., “DSH” hospitals).

Finally, this study demonstrates how the health and human rights framework may be applied as tool for analysis in the ED setting. Although clinical outcomes, cost effectiveness, and patient satisfaction have become well-recognized frameworks for assessing ED interventions, the health and human rights framework is arguably unique from other health-related frameworks (e.g., patients' rights, consumer rights) in that it is grounded in the concept of health equity. Alternative frameworks of analysis can have divergent ramifications in the analysis of a singular reality. As an example, during the HIV/AIDS pandemic of the 1990s, cost-effective analysis that held funding and drug prices to be fixed determined that it would be cost-effective to defer treatment of those already inflicted with HIV in favor of preventative interventions. A health and human rights framework applied to the same reality, in confluence with the rise of generic antiretroviral drugs and grassroots activism, exponentially increased global access to life-saving drugs.^18^

### Limitations

This study has several limitations. The study population was a convenience sample of patients in the ED who were deemed not to be in acute distress. Furthermore, the study was conducted entirely at a single safety-net ED, and, therefore, is not representative of the national population. All demographic characteristics were self-reported and may deviate from actual characteristics. Similarly, self-reported barriers to health and prioritizations maybe biased toward health care given the context of the questionnaire being administered in the ED. Finally, this study was performed shortly after ACA implementation. In the interim, the ACA has improved access to health care and health outcomes for patients covered by its provisions.^19^ Many of our ED's previously uninsured patients have benefited from the ACA; rates of uninsured decreased in Los Angeles County from 28.5% in 2011^20^ to ∼10.5% in 2016,^21^ and nationally from 17.4% to 10.9%.^22^

Nonetheless, barriers to health care and health persist.^23^ Thirty-six percent of this study's subjects were undocumented immigrants, unable to benefit directly from Medicaid expansion or the health insurance exchanges. High and increasing health insurance premiums for plans offered on the exchanges or through other private entities further limit participation; between 2008 and 2018, premiums for family coverage increased to 55%, and employees' share by 65%.^24^ Even among those who have recently acquired health insurance, narrow networks and lack of specialty providers willing to except Medicaid rates limit specialty care access,^25,26^ which was a concern of our 91% of our subjects. However, the ACA has accelerated emphasis on addressing social determinants of health^27,28^ by connecting patients to community resources, food/nutrition, housing, education, employment, and so on. Such focus “upstream”^29^ benefits even those ineligible for ACA-related health insurance,^30^ including our ED population,^31^ among whom such social determinants represented substantial concerns.

## Conclusion

Although the ACA has made notable strides in extending the provision of health insurance in its early stages, this study suggests that barriers to the RTH among marginalized patient populations persist. These barriers can be further divided into barriers to health care and barriers to health. Barriers to health care include the explicit exclusion of individuals from coverage (e.g., the undocumented), structural barriers to the realization of health care services, and underfunding of health institutions to serve the newly insured poor. Despite passage of the ACA barriers to a holistic definition of health also persist in this study population, highlighting the need for collaboration between health providers, patients, and advocates to address structural factors that have significant bearing on health outcomes and equity. Finally, this study demonstrates how the health and human rights framework maybe used as a tool to promote health and health equity among marginalized patient populations.

## Abbreviations Used

ACA: Affordable Care Act
DACA: Deferred Action for Childhood Arrivals
ED: emergency department
FPL: federal poverty level
RTH: right to health
SD: standard deviation
